# Uso do Modelo de Risco ADHERE como Preditor de Piora Intra-Hospitalar de Insuficiência Cardíaca em uma Coorte

**DOI:** 10.36660/abc.20220584

**Published:** 2023-08-10

**Authors:** Daniela de Souza Bernardes, Marina Scherer Santos, Vanessa Monteiro Mantovani, Omar Pereira de Almeida, Livia Adams Goldraich, Nadine Clausell, Eneida Rejane Rabelo-Silva

**Affiliations:** 1 Programa de Pós-Graduação em Ciências da Saúde: Cardiologia e Ciências Cardiovasculares Universidade Federal do Rio Grande do Sul Porto Alegre RS Brasil Programa de Pós-Graduação em Ciências da Saúde: Cardiologia e Ciências Cardiovasculares, Universidade Federal do Rio Grande do Sul, Porto Alegre, RS – Brasil; 2 Escola de Enfermagem Universidade Federal do Rio Grande do Sul Porto Alegre RS Brasil Escola de Enfermagem, Universidade Federal do Rio Grande do Sul, Porto Alegre, RS – Brasil; 3 Departamento de Enfermagem Universidade Federal de Uberlândia Uberlândia MG Brasil Departamento de Enfermagem, Universidade Federal de Uberlândia, Uberlândia, MG – Brasil; 4 Grupo de Insuficiência Cardíaca e Transplante Cardíaco Hospital de Clínicas de Porto Alegre Porto Alegre RS Brasil Divisão de Cardiologia, Grupo de Insuficiência Cardíaca e Transplante Cardíaco, Hospital de Clínicas de Porto Alegre (HCPA), Porto Alegre, RS – Brasil

**Keywords:** Insuficiência Cardíaca, Hospitalização, Grupos de Risco

## Abstract

**Fundamento:**

Pacientes hospitalizados com insuficiência cardíaca (IC) aguda descompensada estão sujeitos a desenvolver episódios de piora que requerem intervenções mais complexas. O modelo de predição de risco “Acute Decompensated Heart Failure National Registry” (ADHERE) foi desenvolvido nos Estados Unidos para prever o risco de piora intra-hospitalar da IC.

**Objetivo:**

Utilizar o modelo de predição de risco ADHERE para avaliar o risco de piora intra-hospitalar da IC e determinar a sua sensibilidade e especificidade em pacientes hospitalizados.

**Métodos:**

O presente estudo de coorte foi realizado em um hospital universitário público brasileiro e os dados de 2013 a 2020 foram coletados retrospectivamente. Foram considerados estatisticamente significativos valores de p < 0,05.

**Resultados:**

Foram incluídos 890 pacientes com idade média de 74 ± 8 anos. O modelo mostrou que no grupo de 490 pacientes de risco, 254 (51,8%) desenvolveram piora intra-hospitalar da IC. No grupo de 400 pacientes sem risco, apenas 109 (27,2%) apresentaram piora da IC. Os resultados demonstraram uma curva estatisticamente significativa (área sob a curva = 0,665; erro padrão = 0,018; p < 0,01; intervalo de confiança = 0,609 a 0,701), indicando boa precisão. O modelo apresentou sensibilidade de 69,9% e especificidade de 55,2%, com valor preditivo positivo de 52% e valor preditivo negativo de 72,7%.

**Conclusões:**

Na presente coorte, demonstramos que o modelo de predição de risco ADHERE foi capaz de discriminar pacientes que, de fato, desenvolveram piora da IC durante o período de internação daqueles que não desenvolveram.

**Figura Central:**
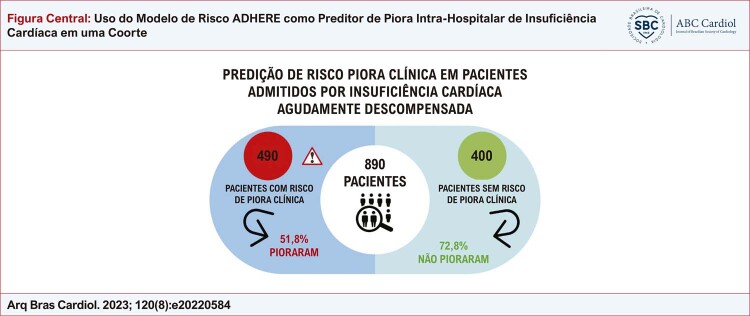
: Uso do Modelo de Risco ADHERE como Preditor de Piora Intra-Hospitalar de Insuficiência Cardíaca em uma Coorte

## Introdução

As internações hospitalares por insuficiência cardíaca (IC) aguda descompensada representam um marco no curso natural da síndrome e constituem um fator de risco relevante para a mortalidade no ano seguinte à internação e para re-hospitalizações recorrentes.^1^ A cada novo episódio de descompensação e internação hospitalar, o prognóstico piora, de modo que aproximadamente 50% dos pacientes que recebem alta são readmitidos nos 12 meses seguintes.^2^

Pacientes hospitalizados com IC aguda descompensada estão sujeitos a desenvolver episódios de piora durante a internação que requerem intervenções mais complexas, por exemplo, medicamentos inotrópicos e/ou vasodilatadores intravenosos, ou mesmo transferência para unidades de terapia intensiva.^3^ Nesse contexto, foi desenvolvido o “Acute Decompensated Heart Failure National Registry” (ADHERE) nos Estados Unidos para prever o risco de piora intra-hospitalar da IC. O ADHERE é considerado o maior registro internacional de IC e foi concebido para possibilitar estudos que avaliam características clínicas, manejo do cuidado e suas implicações em uma grande amostra de pacientes internados por IC.^4^

O ADHERE foi aplicado em um estudo de coorte realizado no Brasil com 634 pacientes hospitalizados. Foram testados parâmetros ecocardiográficos que, associados ao ADHERE, melhorariam sua precisão para avaliar o risco de mortalidade intra-hospitalar. A estimativa do risco de mortalidade usando apenas o ADHERE foi limitada. O parâmetro ecocardiográfico da pressão sistólica da artéria pulmonar teve valor prognóstico independente, aumentando discretamente a precisão do escore.^5^

Subsequentemente, foi realizado um novo estudo para validar o modelo predição de risco ADHERE para prever a piora intra-hospitalar da IC.^6^ Os resultados demonstraram que 37% dos pacientes que estavam com risco de desenvolver piora da IC de acordo com o modelo realmente desenvolveram, enquanto 89% dos pacientes sem risco não apresentaram piora da IC.^6^ Para avaliar o risco de piora da IC para cada paciente, foram incluídas 9 variáveis demográficas, clínicas e laboratoriais no modelo: idade, frequência cardíaca, pressão arterial, fração de ejeção do ventrículo esquerdo (FEVE), peptídeo natriurético tipo B (BNP), troponina, sódio, nitrogênio ureico no sangue e creatinina.^6^

Os resultados do modelo de predição de risco ADHERE permitem que a equipe de saúde avance e planeje intervenções individualizadas com a finalidade de potencialmente minimizar ou diminuir o risco de instabilidade clínica durante a hospitalização. Essa ferramenta ainda não foi testada em uma amostra brasileira de pacientes com IC aguda descompensada, o que surge como uma proposta interessante para a avaliação de sua aplicabilidade. Portanto, o objetivo foi usar o modelo de risco ADHERE para predizer a piora da IC em pacientes hospitalizados e avaliar sua sensibilidade e especificidade. O presente estudo é relevante para a prática clínica, pois agrega mais uma ferramenta que pode auxiliar na tomada de decisões no âmbito hospitalar.

## Métodos

### Desenho e local do estudo

O presente estudo de coorte foi realizado a partir da coleta retrospectiva de dados de pacientes com IC aguda descompensada admitidos na unidade de emergência de um hospital universitário público da Região Sul do Brasil no período de 2013 a 2020. O hospital atende cerca de 60 especialidades médicas e é referência para transplante cardíaco. O estudo foi aprovado pelo Comitê de Ética em Pesquisa Institucional, de acordo com a Resolução 466/2012.

### Participantes

O estudo incluiu homens e mulheres com idade ≥ 60 anos, hospitalizados com IC aguda descompensada, cujos resultados do exame de BNP estavam disponíveis na admissão. Foram excluídos pacientes cuja internação foi eletiva e/ou que necessitaram do uso de drogas vasoativas para o manejo do episódio de IC aguda descompensada (medicamentos inotrópicos e/ou vasodilatadores intravenosos) no momento da admissão ou que foram transferidos para unidades de terapia intensiva nas primeiras 12 horas de hospitalização. Foram identificados os pacientes elegíveis por meio de uma busca com filtros relacionados ao código I50 da CID-10 e unidades como o pronto-socorro, a unidade intensiva de cardiologia e a enfermaria clínica.

### Variáveis e fonte de dados

Foi elaborada uma lista de pacientes respeitando os critérios de inclusão e exclusão. A partir dessa lista, foram coletados retrospectivamente os dados de admissão dos pacientes (variáveis clínicas, laboratoriais e demográficas): idade e história médica prévia (hipertensão, diabetes mellitus, fibrilação atrial, dislipidemia, doença arterial coronariana, doença renal crônica, doença pulmonar obstrutiva crônica, hipotireoidismo, acidente vascular cerebral e história de tabagismo). Também foram consultados os prontuários eletrônicos para dados faltantes.

Foram usadas as 9 variáveis do modelo de risco ADHERE (idade, frequência cardíaca, pressão arterial sistólica, FEVE, BNP, troponina, sódio, nitrogênio ureico no sangue e creatinina) para prever o risco de piora da IC. Em seguida, foi calculada uma pontuação para cada paciente.^6^

Com base nas pontuações, a amostra foi dividida em dois grupos: a) grupo de risco para desenvolver piora intra-hospitalar da IC; b) grupo sem risco de desenvolver piora intra-hospitalar da IC. Foi realizada a análise dos dados de internação hospitalar do prontuário eletrônico com o objetivo de verificar se cada grupo apresentou piora da IC de acordo com o modelo de predição de risco.

Os critérios de piora da IC intra-hospitalar foram: necessidade de medicação inotrópica e vasodilatadora > 12 horas após a admissão, ventilação mecânica, hemodiálise, suporte circulatório mecânico e transferência para unidades de cuidado intensivo. A presença de qualquer um desses critérios, isoladamente ou combinados, foi considerada piora da IC. O modelo de predição de risco foi calculado usando a fórmula do estudo original. Esse cálculo é baseado na análise de regressão logística para predizer o risco de piora intra-hospitalar da IC, utilizando como ponto de corte o valor de 15%, ou seja, valores de probabilidade predita abaixo de 0,2559150, obtidos pelo cálculo da pontuação de risco.^6^

### Análise estatística

Todas as variáveis contínuas apresentaram distribuição normal, de acordo com o resultado do teste de normalidade de Shapiro-Wilk; portanto, foram expressos em média e desvio padrão. As variáveis categóricas foram descritas como frequências absolutas e relativas. Foi utilizado o teste t de Student não pareado para comparar dois grupos de variáveis contínuas. A associação de variáveis categóricas entre os dois grupos de risco foi analisada pelo teste qui-quadrado. Foi utilizado o modelo de análise de regressão logística para predizer o risco de piora intra-hospitalar. A análise da área sob a curva (AUC) foi realizada para avaliar a precisão do modelo.

Foi avaliada a mortalidade intra-hospitalar pela regressão de Cox de acordo com o grupo de risco e o modelo foi ajustado para a variável sexo. Todas as análises estatísticas foram realizadas no SPSS 18.0 e foram considerados estatisticamente significativos valores de p < 0,05.

## Resultados

Em total, foram incluídos 890 pacientes. Destes, o modelo de risco ADHERE colocou 490 (55%) pacientes no grupo de risco de piora intra-hospitalar da IC e 400 (45%) pacientes ao grupo sem risco de piora intra-hospitalar da IC. A Tabela 1 exibe as variáveis do modelo e as características demográficas e clínicas da amostra. A média de idade foi semelhante nos dois grupos. Sexo, diabetes mellitus, doença renal crônica e doença pulmonar obstrutiva crônica foram significativos (p < 0,01) e mais frequentes no grupo de risco quando comparado ao grupo sem risco.

**Tabela 1:** – Variáveis do modelo e características de pacientes com insuficiência cardíaca aguda descompensada

Variáveis	Com risco (490)	Sem risco (400)	p	OR	IC 95%
Idade*	72 ± 8	77 ± 8	--	--	--
Pressão arterial sistólica*	126 ± 30	132 ± 24	--	--	--
Pulso*	89 ± 21	83 ± 22	--	--	--
Fração de ejeção do ventrículo esquerdo*	37 ± 16	47 ± 16	--	--	--
Peptídeo natriurético tipo B*	1384 ± 1317	382 ± 326	--	--	--
Troponina*	0,110 ± 0,174	0,026 ± 0,083	--	--	--
Sódio*	138 ± 8	140 ± 4	--	--	--
Nitrogênio ureico no sangue*	94 ± 50	61 ± 29	--	--	--
Creatinina*	2,02 ± 1,74	1,24 ± 0,44	--	--	--
**Sexo** ^**†**^					
Masculino	303 (61,8)	197 (49,3)	0,01	1,66	1,27-2,18
Feminino	187 (38,2)	203 (50,8)	--	--	--
**Cor da pele^**†**^**					
Branca	433 (88,4)	356 (89,0)	--	--	--
Parda	48 (9,8)	35 (8,8)	0,79	0,45	0,77-0,79
Preta	9 (1,8)	9 (2,3)	--	--	--
**Comorbidades^**†**^**					
Hipertensão	361 (73,7)	310 (77,5)	0,21	1,23	0,90-1,67
Diabetes	245 (50,0)	166 (41,5)	0,01	0,70	0,54-0,92
Fibrilação	199 (40,6)	177 (44,3)	0,27	1,16	0,88-1,51
Dislipidemia	38 (7,8)	38 (9,5)	0,39	1,24	0,78-1,99
Doença arterial coronariana	136 (27,8)	99 (24,8)	0,32	0,85	0,63-1,15
Doença renal crônica	185 (37,8)	67 (16,8)	0,01	0,33	0,24-0,45
Doença pulmonar obstrutiva crônica	84 (17,1)	97 (24,3)	0,01	1,54	1,15-2,14
Hipotireoidismo	58 (11,8)	58 (14,5)	0,27	1,26	0,85-1,86
Acidente vascular cerebral	86 (17,5)	65 (16,3)	0,65	0,91	0,64-1,30
Tabagismo	39 (8,0)	38 (9,5)	0,47	1,21	0,76-1,93

*Variável contínua expressa em média e desvio padrão (±); ^†^variável categórica expressa em número e porcentagens (%). IC: intervalo de confiança; OR: odds ratio. Fonte: Dados da pesquisa, 2022.

No grupo de 490 pacientes de risco, 254 (51,8%) desenvolveram piora intra-hospitalar da IC, enquanto no grupo de 400 pacientes sem risco, 291 (72,8%) não pioraram. Em relação aos desfechos de piora intra-hospitalar, todos os critérios apresentaram diferença significativa em ambos os grupos, com exceção do uso de ventilação mecânica. Para o cálculo da *odds ratio* , o grupo sem risco foi considerado o grupo de referência. O uso de vasodilatadores foi mais prevalente que os demais critérios, de acordo com os dados apresentados na Tabela 2 .

**Tabela 2:** – Desfechos da piora intra-hospitalar da insuficiência cardíaca nos grupos de risco e sem risco

Critérios de piora da insuficiência cardíaca	Com risco (490) N (%)	Sem risco (400) N (%)	p	OR	IC 95%
Vasodilatador > 12 horas após a admissão	188 (38)	79 (20)	< 0,001	1,74	1,43-2,13
Medicamento inotrópico > 12 horas após a admissão	96 (19)	32 (8)	< 0,001	1,93	1,42-2,63
Ventilação mecânica	42 (9)	55 (14)	0,014	0,76	0,63-0,93
Hemodiálise	62 (13)	7 (2)	< 0,001	4,72	2,33-9,56
Suporte circulatório mecânico	25 (5)	1(0,3)	< 0,001	12,0	1,75-82,2

*Variável categórica expressa em número (%). IC: intervalo de confiança; OR: odds ratio. Fonte: Dados da pesquisa, 2022.

**Figura 1:**
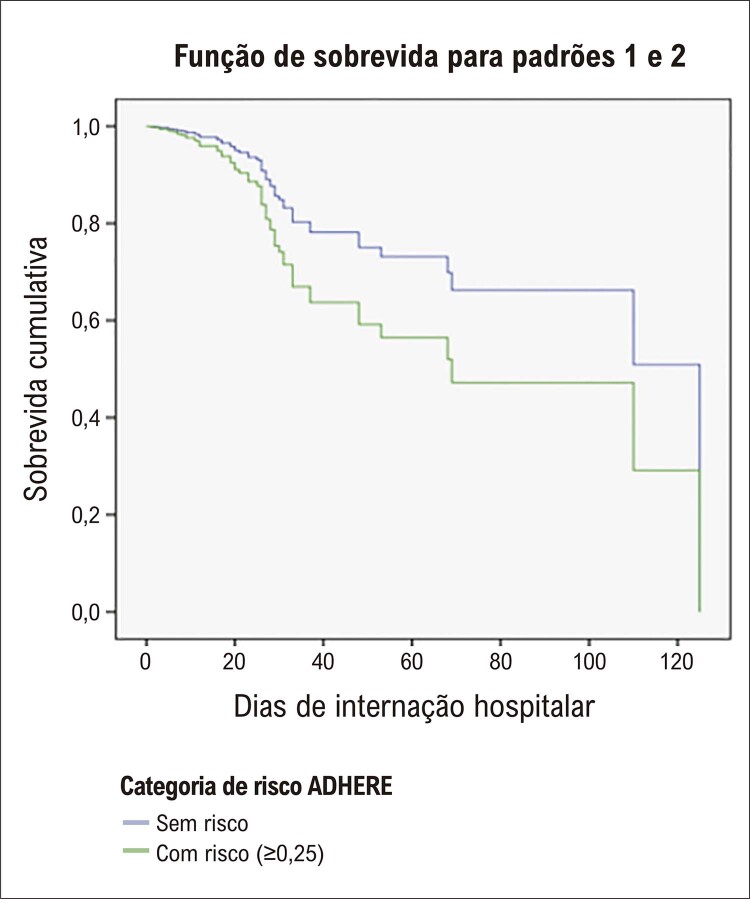
– Análise de sobrevida por regressão de Cox. Fonte: Dados da pesquisa, 2022.

A análise de sobrevida foi realizada por meio da regressão de Cox, onde foi observada mortalidade intra-hospitalar de acordo com a classificação de risco (p = 0,063), em um modelo ajustado por sexo, conforme demonstrado na Figura 1 . Durante o período de acompanhamento hospitalar (média do tempo de internação de 13 dias), ocorreram 56 óbitos (6,3%). De acordo com a classificação de risco do ADHERE, não houve diferença na mortalidade intra-hospitalar ( *hazard ratio* : 1,82; intervalo de confiança de 95%: 0,97 a 3,43; p = 0,063).

**Figura 2:**
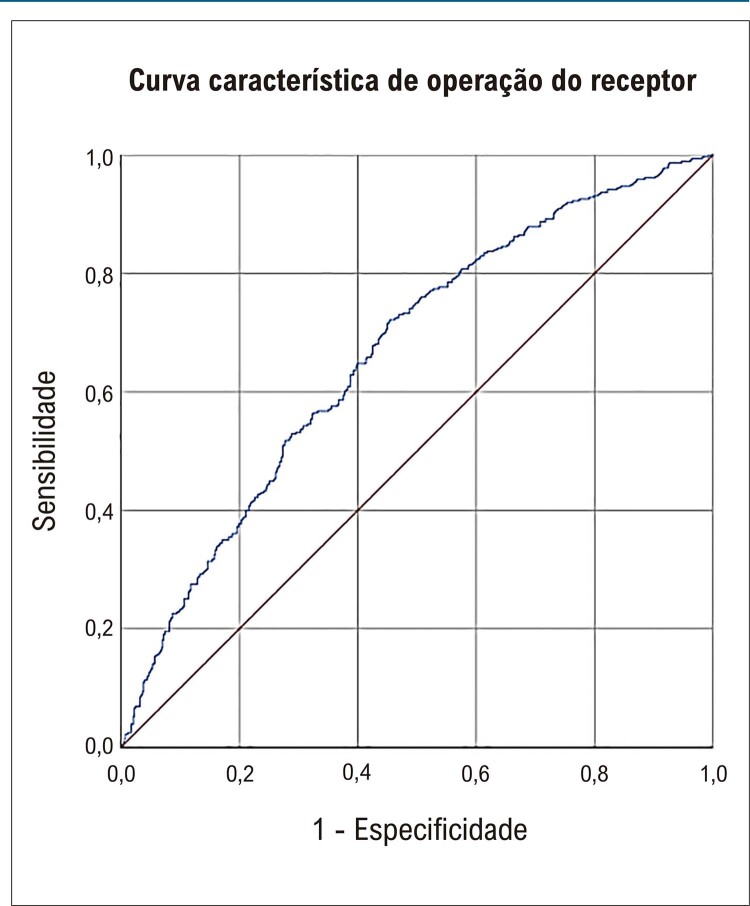
– Análise de sensibilidade e especificidade de pacientes com insuficiência cardíaca aguda descompensada. Fonte: Dados da pesquisa, 2022.

Conforme mostrado na Figura 2 , para avaliar a precisão do modelo ADHERE para risco de piora da IC, os resultados demonstraram uma curva estatisticamente significativa (AUC = 0,665; erro padrão = 0,018; p < 0,01; intervalo de confiança = 0,609 a 0,701), indicando boa precisão. O modelo de risco ADHERE apresentou sensibilidade de 69,9% e especificidade de 55,2%, com valor preditivo positivo de 52% e valor preditivo negativo de 72,7%.

## Discussão

Não há descrição prévia na literatura científica de outros estudos que utilizaram escores para avaliar o prognóstico admissional no âmbito de piora intra-hospitalar da IC em centros da América Latina. Em contraste, a diretriz brasileira para IC aguda recomenda (classe de recomendação I e nível de evidência A) o uso de escores de estratificação de risco intra-hospitalar no momento da admissão.^7 , 8^ Diretrizes internacionais também recomendam o uso de escores de estratificação de risco intra-hospitalar no momento da admissão.^6 - 10^

Os achados do presente estudo foram semelhantes aos do estudo original que avaliou o modelo de predição de risco ADHERE para piora da IC, uma vez que aproximadamente metade dos pacientes de risco realmente apresentou piora intra-hospitalar da IC. A maioria dos pacientes sem risco não desenvolveu piora da IC.

Em relação aos critérios de piora da IC, medicamentos vasodilatadores e inotrópicos foram usados em 30% e 14% da amostra total, respectivamente, > 12 horas após a admissão. No Registro Brasileiro de Insuficiência Cardíaca Aguda (BREATHE), menos de 15% da amostra recebeu essas terapias intravenosas.^11 , 12^ Esses medicamentos são indicados pelas atuais diretrizes de IC aguda porque atuam controlando os sintomas e corrigindo distúrbios hemodinâmicos, como a redução do débito cardíaco e o aumento das pressões de enchimento.^9 , 13 , 14^

De acordo com as diretrizes brasileiras para IC aguda, o uso de vasodilatadores intravenosos no âmbito de IC aguda é considerado classe de recomendação I e nível de evidência B.^1^ Vasodilatadores como nitroprussiato de sódio e nitroglicerina são altamente indicados para alívio da congestão pulmonar, pois aumentam o débito e, portanto, a diurese, sendo importantes para controlar a pressão arterial em pacientes com hipertensão e melhorar a dispnéia.^9 , 14^ Embora os medicamentos inotrópicos estejam associados a um aumento de isquemia e arritmias, eles não são tão eficazes para os desfechos hemodinâmicos.^14 - 17^

No presente estudo, 11% da amostra necessitou de ventilação mecânica, 8% de terapia dialítica e 3% de suporte circulatório mecânico. Tais fatores são típicos da piora da IC, embora a sua definição permaneça controversa na literatura.^18 - 20^ De modo geral, essa condição é caracterizada por aumento de diuréticos (por exemplo, aumento de dose, adição de um diurético tiazídico a diuréticos de alça ou reinício de terapia intravenosa); início de medicamentos inotrópicos, vasopressores ou vasodilatadores intravenosos; e uso de ventilação mecânica ou suporte circulatório.^4^

Embora o uso de ventilação mecânica tenha sido relativamente baixo (11%) no presente estudo, sabe-se que pacientes com IC aguda descompensada apresentam múltiplas morbidades, inclusive não cardíacas, desencadeando complicações respiratórias.^17^ Um estudo anterior mostrou uma incidência de ventilação mecânica que variava de 5,0% a 13,9% em pacientes internados com IC aguda descompensada. No entanto, a relação entre o uso de estratégias de suporte ventilatório em pacientes com disfunção ventricular e como a insuficiência respiratória afeta os resultados clínicos ainda não está clara na literatura.^8 - 13^ O suporte ventilatório invasivo é considerado para pacientes com IC aguda descompensada que permanecem sintomáticos e/ou hipoxêmicos apesar de outras formas não invasivas de suporte ventilatório.^8^

Uma pequena parcela da amostra (8%) necessitou de hemodiálise. Um estudo anterior constatou que pacientes com IC aguda descompensada apresentaram melhora significativa da congestão com o início da ultrafiltração venovenosa. No entanto, essa intervenção clínica deve ser ajustada de acordo com as necessidades individuais de cada paciente, pois pode paradoxalmente induzir uma piora da função renal, sem quaisquer benefícios clínicos.^15^

O prognóstico admissional da IC aguda descompensada permite um planejamento terapêutico precoce, proporcionando intervenções específicas e individualizadas para minimizar o risco previsto da piora intra-hospitalar da IC e permitindo o acompanhamento dos pacientes com protocolos e unidades terapêuticas mais adequados.

A coleta retrospectiva de dados e o fato de ser um estudo unicêntrico são limitações do presente estudo, o que pode restringir a aplicação e a análise de escores de predição de risco a coortes diferentes.

## Conclusões

Nossos resultados demonstram que a aplicação do ADHERE na admissão hospitalar pode melhorar a previsão de risco de piora da IC. Nesta coorte de pacientes com IC aguda descompensada, o escore de risco apresentou maior sensibilidade do que especificidade. No entanto, devido à sua capacidade limitada de predizer a piora da IC, não deve ser utilizado isoladamente na prática clínica. Mais estudos multicêntricos são necessários para investigar outras possíveis aplicações desses achados.
