# Supplementary material for: Uso do Modelo de Risco ADHERE como Preditor de Piora Intra-Hospitalar de Insuficiência Cardíaca em uma Coorte
Source: Arq Bras Cardiol. 2023 Aug 10;120(8):e20220584. [Article in Portuguese] doi: 10.36660/abc.20220584 (PMC10519228; doi:10.36660/abc.20220584)
Supplement: Translation

# Translation: Use of the ADHERE Risk Model as a Predictor of Risk of in-Hospital Worsening Heart Failure in a Cohort

**Authors:** Daniela de Souza Bernardes, Marina Scherer Santos, Vanessa Monteiro Mantovani, Omar Pereira de Almeida, Livia Adams Goldraich, Nadine Clausell, Eneida Rejane Rabelo-Silva

**Affiliations:** 1 Brasil Programa de Pós-Graduação em Ciências da Saúde: Cardiologia e Ciências Cardiovasculares, Universidade Federal do Rio Grande do Sul, Porto Alegre, RS – Brasil; 2 Brasil Escola de Enfermagem, Universidade Federal do Rio Grande do Sul, Porto Alegre, RS – Brasil; 3 Brasil Departamento de Enfermagem, Universidade Federal de Uberlândia, Uberlândia, MG – Brasil; 4 Brasil Divisão de Cardiologia, Grupo de Insuficiência Cardíaca e Transplante Cardíaco, Hospital de Clínicas de Porto Alegre (HCPA), Porto Alegre, RS – Brasil

**Keywords:** Heart Failure, Hospitalization, Risk Groups

## Abstract

**Background:**

Patients admitted with acute decompensated heart failure (HF) are subject to developing worsening episodes that require more complex interventions. The Acute Decompensated Heart Failure National Registry (ADHERE) risk model was developed in the United States to predict the risk of in-hospital worsening HF.

**Objective:**

To use the ADHERE risk model in the assessment of risk of in-hospital worsening HF and to determine its sensitivity and specificity in hospitalized patients.

**Methods:**

This cohort study was conducted at a Brazilian public university hospital, and data from 2013 to 2020 were retrospectively collected. P values < 0.05 were considered statistically significant.

**Results:**

A total of 890 patients with a mean age of 74 ± 8 years were included. The model showed that, in the group of 490 patients at risk, 254 (51.8%) developed in-hospital worsening HF. In the group of 400 patients not at risk, only 109 (27.2%) experienced worsening HF. The results demonstrated a statistically significant curve (area under the curve = 0.665; standard error = 0.018; P < 0.01; confidence interval = 0.609 to 0.701), indicating good accuracy. The model had a sensitivity of 69.9% and a specificity of 55.2%, with a positive predictive value of 52% and a negative predictive value of 72.7%.

**Conclusions:**

In this cohort, we showed that the ADHERE risk model was able to discriminate patients who in fact developed worsening HF during the admission period, from those who did not.

**Central Illustration:** : Use of the ADHERE Risk Model as a Predictor of Risk of in-Hospital Worsening Heart Failure in a Cohort

## Introduction

Hospital admissions for acute decompensated heart failure (HF) represent a milestone in the natural course of this syndrome and constitute a relevant risk factor for mortality during the year following hospitalization and for recurrent readmissions.^1^With each new episode of decompensation and admission, the prognosis becomes poorer, so that approximately 50% of discharged patients are readmitted within the following 12 months.^2^

Patients admitted with acute decompensated HF are subject to developing worsening episodes during hospitalization that require more complex interventions, such as inotropic medications and/or intravenous vasodilators, or even transfer to intensive care units.^3^Within this context, the Acute Decompensated Heart Failure National Registry (ADHERE) was developed in the United States. The ADHERE is considered the largest HF international registry, and was designed to enable studies that assess clinical characteristics, management of care, and their implications in a large sample of patients hospitalized for HF.^4^

The ADHERE was applied in a cohort study that took place in Brazil with 634 hospitalized patients. Echocardiographic parameters that, associated with the ADHERE, would improve its accuracy to assess the risk of hospital mortality were tested. Estimation of mortality risk using the ADHERE alone was limited. The echocardiographic parameter pulmonary artery systolic pressure had independent prognostic value, slightly increasing the score’s accuracy.^5^

Subsequently, a new study was performed to validate the ADHERE risk model for predicting in-hospital worsening HF.^6^The results showed that 37% of patients who were at risk of developing worsening HF according to the model actually developed it, while 89% of patients who were not at risk did not experience worsening HF.^6^To assess the risk of worsening HF for each patient, 9 demographic, clinical, and laboratory variables were included in the model: age, heart rate, blood pressure, left ventricular ejection fraction (LVEF), B-type natriuretic peptide (BNP), troponin, sodium, blood urea nitrogen, and creatinine.^6^

The ADHERE risk model results allow the health care team to advance and plan individualized interventions in order to potentially minimize or decrease the risk of clinical instability during hospitalization. This tool has not yet been tested in a Brazilian sample of patients with acute decompensated HF, which emerges as an interesting proposal for evaluating its applicability. Therefore, the aim was to use the ADHERE risk model for predicting worsening HF in hospitalized patients and to assess its sensitivity and specificity. This study is relevant to clinical practice as it adds another tool that can assist in decision-making within hospital settings.

## Methods

### Study design and setting

This cohort study was based on retrospective data collection of patients with acute decompensated HF admitted to the emergency department of a public university hospital in the South Region of Brazil from 2013 to 2020. This hospital provides assistance in about 60 medical specialties and is a reference for heart transplantation. The study was approved by the Institutional Research Ethics Committee, in accordance with Brazilian Resolution No. 466/2012.

### Participants

The study included men and women aged ≥ 60 years, hospitalized with acute decompensated HF, whose BNP test results were available at admission. Patients whose admission was elective and/or who required the use of vasoactive drugs for the management of the acute decompensated HF episode (inotropic medications and/or intravenous vasodilators) at the time of admission or who were transferred to intensive care units within the first 12 hours of hospitalization were excluded. Eligible patients were identified by running a query using filters related to ICD-10 code I50 and units such as the emergency room, cardiology intensive unit, and clinical ward.

### Variables and data source

A list of patients was prepared respecting the inclusion and exclusion criteria. From this list, patient admission data (clinical, laboratory, and demographic variables) were collected retrospectively: age and previous medical history (hypertension, diabetes mellitus, atrial fibrillation, dyslipidemia, coronary artery disease, chronic kidney disease, chronic obstructive pulmonary disease, hypothyroidism, stroke, and history of smoking). Electronic medical records were also consulted for missing data.

The 9 ADHERE risk model variables (age, heart rate, systolic blood pressure, LVEF, BNP, troponin, sodium, blood urea nitrogen, and creatinine) were used to predict the risk of worsening HF. Then, a score was calculated for each patient.^6^

Based on the scores, the sample was divided into two groups: a) group at risk of developing in-hospital worsening HF; b) group not at risk of developing in-hospital worsening HF. The analysis of hospital stay data from the electronic medical records aimed to ascertain whether each group had worsening HF according to the risk prediction model.

In-hospital worsening HF criteria were the need for vasodilator or inotropic medication > 12 hours after admission, mechanical ventilation, hemodialysis or mechanical circulatory support, and transfer to intensive care units. The presence of any of these criteria, alone or combined, was considered worsening HF. The risk prediction model was calculated using the formula from the original study. This calculation is based on logistic regression analysis for predicting the risk of in-hospital worsening HF using a cutoff point of 15%, that is, predicted probability values below 0.2559150, obtained through calculation of the risk score.^6^

### Statistical analysis

All continuous variables presented normal distribution, according to the result of the Shapiro-Wilk normality test; therefore, they were expressed as mean and standard deviation. Categorical variables were described as absolute and relative frequencies. Unpaired Student’s t test was used to compare two groups of continuous variables. The association of categorical variables between the two risk groups was analyzed by the chi-square test. The logistic regression analysis model was used to predict the risk of in-hospital worsening. AUC (area under the curve) analysis was performed to assess the accuracy of the score.

In-hospital mortality was assessed using Cox regression according to the risk group, and the model was adjusted for the variable of sex. All statistical analyses were performed using SPSS 18.0, and p values < 0.05 were considered statistically significant.

## Results

In total, 890 patients were included. Of these, the ADHERE risk model assigned 490 (55%) patients to the group at risk of developing in-hospital worsening HF and 400 (45%) patients to the group not at risk of worsening HF. Table 1 shows score variables and demographic and clinical characteristics of the sample. Mean age was similar in both groups. Sex, diabetes mellitus, chronic kidney disease, and chronic obstructive pulmonary disease were significant (p < 0.01) and more frequent in the at-risk group when compared to the no-risk group.

**Table 1:** – Score variables and characteristics of patients with acute decompensated heart failure

Variables	At risk (490)	No risk (400)	p	OR	95% CI
Age*	72 ± 8	77 ± 8	--	--	--
Systolic blood pressure*	126 ± 30	132 ± 24	--	--	--
Pulse*	89 ± 21	83 ± 22	--	--	--
Left ventricular ejection fraction*	37 ± 16	47 ± 16	--	--	--
B-type natriuretic peptide*	1384 ± 1317	382 ± 326	--	--	--
Troponin*	0.110 ± 0.174	0.026 ± 0.083	--	--	--
Sodium*	138 ± 8	140 ± 4	--	--	--
Blood urea nitrogen*	94 ± 50	61 ± 29	--	--	--
Creatinine*	2.02 ± 1.74	1.24 ± 0.44	--	--	--
**Sex** ^**†**^					
Male	303 (61.8)	197 (49.3)	0.01	1.66	1.27-2.18
Female	187 (38.2)	203 (50.8)	--	--	--
**Skin color^**†**^**					
White	433 (88.4)	356 (89.0)	--	--	--
Brown	48 (9.8)	35 (8.8)	0.79	0.45	0.77-0.79
Black	9 (1.8)	9 (2.3)	--	--	--
**Comorbidities^**†**^**					
Hypertension	361 (73.7)	310 (77.5)	0.21	1.23	0.90-1.67
Diabetes	245 (50.0)	166 (41.5)	0.01	0.70	0.54-0.92
Fibrillation	199 (40.6)	177 (44.3)	0.27	1.16	0.88-1.51
Dyslipidemia	38 (7.8)	38 (9.5)	0.39	1.24	0.78-1.99
Coronary artery disease	136 (27.8)	99 (24.8)	0.32	0.85	0.63-1.15
Chronic kidney disease	185 (37.8)	67 (16.8)	0.01	0.33	0.24-0.45
Chronic obstructive pulmonary disease	84 (17.1)	97 (24.3)	0.01	1.54	1.15-2.14
Hypothyroidism	58 (11.8)	58 (14.5)	0.27	1.26	0.85-1.86
Stroke	86 (17.5)	65 (16.3)	0.65	0.91	0.64-1.30
Smoking	39 (8.0)	38 (9.5)	0.47	1.21	0.76-1.93

*Continuous variable expressed as mean and standard deviation (±);^†^categorical variable expressed as n and percentages (%). CI: confidence interval; OR: odds ratio. Source: Research data, 2022.

In the group of 490 patients at risk, 254 (51.8%) developed in-hospital worsening HF, whereas, in the group of 400 patients without risk, 291 (72.8%) did not worsen. Regarding the in-hospital worsening outcomes, all criteria presented significant difference in both groups, with the exception of mechanical ventilation use. For calculation of the odds ratio, the group without risk was considered the reference group. The use of vasodilators was more prevalent than the other criteria, according to data presented in Table 2 .

**Table 2:** – Outcomes of in-hospital worsening heart failure in the at risk and no risk groups

Worsening heart failure criteria	At risk (490) N (%)	No risk (400) N (%)	p	OR	95% CI
Vasodilator > 12 hours after admission	188 (38)	79 (20)	< 0,001	1,74	1,43-2,13
Inotropic medication > 12 hours after admission	96 (19)	32 (8)	< 0,001	1,93	1,42-2,63
Mechanical ventilation	42 (9)	55 (14)	0,014	0,76	0,63-0,93
Hemodialysis	62 (13)	7 (2)	< 0,001	4,72	2,33-9,56
Mechanical circulatory support	25 (5)	1(0,3)	< 0,001	12,0	1,75-82,2

*Categorical variable expressed as n (%). CI: confidence interval; OR: odds ratio. Source: Research data, 2022.

Survival analysis was performed using Cox regression, where in-hospital mortality was observed according to the risk classification (p = 0.063), in a model adjusted for sex, as demonstrated in Figure 1 . During the in-hospital follow-up period (mean length of stay 13 days), there were 56 deaths (6.3%). According to the ADHERE risk categorization, there was no difference in in-hospital mortality (hazard ratio = 1.82; 95% confidence interval = 0.97 to 3.43; p = 0.063).

**Figure 1:** – Survival analysis using Cox regression. Source: Research data, 2022.

As shown in Figure 2 , to assess the accuracy of the ADHERE risk model for risk of worsening HF, the results demonstrated a statistically significant curve (AUC = 0.665; standard error = 0.018; p < 0.01; confidence interval = 0.609 to 0.701), indicating good accuracy. The ADHERE risk model had a sensitivity of 69.9% and a specificity of 55.2%, with a positive predictive value of 52% and a negative predictive value of 72.7%.

**Figure 2:** – Sensitivity and specificity analysis of patients with acute decompensated heart failure. Source: Research data, 2022.

## Discussion

There is no previous description in the scientific literature of other studies that used scores to assess prognosis at admission in the setting of in-hospital worsening HF in Latin American centers. In contrast, the Brazilian guideline for acute HF recommends (class of recommendation I and level of evidence A) the use of in-hospital risk stratification scores at the time of admission.^7 , 8^International guidelines also recommend the use of in-hospital risk stratification scores at the time of admission.^6 - 10^

The findings of the present study were similar to those of the original study that evaluated the ADHERE risk model for worsening HF, since approximately half of the patients at risk actually experienced in-hospital worsening HF. Most patients not at risk did not develop worsening HF.

Regarding worsening HF criteria, vasodilators and inotropic medications were used in 30% and 14% of the total sample, respectively, > 12 hours after admission. In the Brazilian Registry of Acute Heart Failure (BREATHE), less than 15% of the sample received those intravenous therapies.^11 , 12^These drugs are indicated by current acute HF guidelines because they act by controlling symptoms and correcting hemodynamic disorders, such as reduced cardiac output and increased filling pressures.^9 , 13 , 14^

According to the Brazilian guidelines for acute HF, intravenous vasodilator use in the setting of acute HF is considered class of recommendation I and level of evidence B.^1^Vasodilators such as sodium nitroprusside and nitroglycerin are highly indicated for pulmonary congestion relief, as they increase cardiac output and, therefore, diuresis, making them important for controlling blood pressure in patients with hypertension and improving dyspnea.^9 , 14^While inotropic medications are associated with an increase in ischemia and arrhythmias, they are not as effective for hemodynamic outcomes.^14 - 17^

In this study, 11% of the sample required mechanical ventilation, 8% dialysis therapy, and 3% mechanical circulatory support. Such factors are typical of worsening HF, although its definition remains controversial in the literature.^18 - 20^In general, this condition is characterized by an increase in diuretics (e.g., increase in dose, addition of a thiazide diuretic to loop diuretics, or reinstitution of intravenous therapy); initiation of inotropic medications, vasopressors, or intravenous vasodilators; and use of mechanical ventilatory or circulatory support.^4^

Although mechanical ventilation use was relatively low (11%) in this study, it is known that patients with acute decompensated HF have multiple morbidities, including noncardiac ones, triggering respiratory complications.^17^A previous study showed an incidence of mechanical ventilation ranging from 5.0% to 13.9% in patients hospitalized with acute decompensated HF. However, the relationship between the use of ventilatory support strategies in patients with ventricular dysfunction and how respiratory failure affects clinical outcomes is still unclear in the literature.^8 - 13^Invasive ventilatory support is considered in patients with acute decompensated HF who remain symptomatic and/or hypoxemic despite other noninvasive forms of ventilatory support.^8^

A small portion of the sample (8%) required hemodialysis. A previous study found that patients with acute decompensated HF showed a significant improvement in congestion with initiation of venovenous ultrafiltration. However, this clinical intervention should be adjusted according to the individual needs of each patient, as it can paradoxically induce worsening renal function, without any clinical benefits.^15^

Prognosis of acute decompensated HF at admission allows for early therapeutic planning, providing specific and individualized interventions to minimize the predicted risk of in-hospital worsening HF and allowing patients to be followed up by more appropriate protocols and therapeutic units.

Retrospective data collection and being a single-center study are limitations of this study, which may restrict the application and analysis of risk prediction scores to different cohorts.

## Conclusions

Our findings demonstrate that applying the ADHERE at hospital admission may improve risk prediction of worsening HF. In this cohort of patients with acute decompensated HF, the risk score had greater sensitivity than specificity. However, due to its limited capacity to predict worsening HF, it should not be used alone in clinical practice. More multicenter studies are needed to investigate other possible applications of these findings.
